# Emerging Technologies and Novel Innovations in Microfluidics for Accelerating Biomedical Research

**DOI:** 10.3390/mi17101186

**Published:** 2026-10-13

**Authors:** Ke Chen, Nan Xiang, Zhonghua Ni

**Affiliations:** School of Mechanical Engineering, and Jiangsu Key Laboratory for Design and Manufacturing of Precision Medicine Equipment, Southeast University, Nanjing 211189, China; seuchenke@seu.edu.cn

Microfluidics offers an enabling tool for chemical sciences, biomedical research, clinical diagnosis, and even mechanical and information engineering [1,2]. In microfluidics, fluids and biological/industrial particles are manipulated with high precision within miniaturized networks fabricated through lithographic micromachining or other prototyping methods [3]. By exploiting laminar flow, precise spatiotemporal control, and minimal reagent consumption, microfluidics allows various traditional laboratory operating procedures or microelectronic circuits to be integrated on a chip with exceptional resolution and performances [4]. Over the past several decades, the field has grown into a highly interdisciplinary area, contributing to the development of chemical sciences, biomedical research, and information technologies. In biomedical research, microfluidics has become indispensable for sample processing, single-cell analysis, molecular detection, drug testing, and so on [5]. For instance, strategies for isolating and detecting rare cell populations—such as circulating tumor cells or fetal nucleated erythrocytes—from samples with complex cellular backgrounds have provided valuable tools for liquid biopsy in oncology and non-invasive prenatal testing [6,7,8]. Microfluidics has also facilitated the development of rapid, inexpensive, and user-friendly point-of-care testing devices, which are critical for early diagnosis and transmission control during infectious disease outbreaks [9]. As modern science advances, the integration of microfluidics with artificial intelligence, the Internet of Things, and quantum sensing is expected to unlock new functionalities and improved performance [10,11]. Notably, a growing number of novel microfluidic devices and instruments have been commercialized, thereby reshaping biomedical research and clinical practice [12].

We believe that microfluidics will continue to drive transformative advances in biomedical discovery and healthcare. Our Special Issue, entitled “Microfluidics in Biomedical Research,” aims to showcase the most recent progress and innovations in this rapidly evolving area. We invite original research and review articles from researchers across disciplines that address these cutting-edge themes. Topics of interest include, but are not limited to, artificial intelligence (AI) for microfluidic systems; new functions enabled by microfluidics; innovative fabrication methods; fundamental principles underlying biomedical microfluidics; biomedical instruments and point-of-care testing devices based on microfluidics; and emerging applications of microfluidics in biomedical research. By bringing together diverse contributions, this Special Issue seeks to illuminate future directions and foster new collaborations in the field. A total of 14 outstanding papers (including nine Articles, one Communication, one Perspective, and three Reviews) were published in our Special Issue. Next, we will briefly introduce these contributions.

Tayeb et al. (contribution 1) performed three-dimensional (3D) numerical simulations to examine steady laminar flow and thermal mixing behavior of non-Newtonian Al_2_O_3_ nanofluids in a two-layer cross-channel micromixer. The analysis spanned Reynolds numbers from 0.1 to 50 and considered secondary flow structures and thermal mixing performances at two different inlet temperatures. They also assessed how fluid properties and nanoparticle loadings (1–5%) affect mixing effectiveness and entropy generation. Magnetohydrodynamic effects were found to improve mixing by inducing vortices and modifying the flow behaviors. The best performance occurred at 4–5% nanoparticle concentration, Reynolds numbers of 10–25, and Hartmann numbers of 200–300. Mixing energy consumption and the thermal mixing index were also evaluated under various operating conditions. The work offered design guidance for microfluidic systems in practical thermal and mixing applications.

Neubert et al. (contribution 2) developed a 3D-printed microfluidic platform that integrated fluidic breakers and phaseguide features to achieve highly efficient passive mixing. The device contained four comparable channel configurations: a staggered herringbone mixer (SHM), a Tesla mixer, a split-and-recombine (SAR) mixer, and a basic unperturbed channel. To permit bubble-free and parallel priming, a phaseguide-based T-shaped stop structure was positioned at the Y-shaped entrance. Mixing performance was quantified through optical gray-level analysis using two dyes with poor mutual miscibility at flow rates spanning 1–200 μL/min. The uniform SHM produced the greatest mixing across the entire flow range, although a central region remained inadequately mixed at higher flow rates. In a non-uniform SHM layout, fluid breakers separating two SHM segments diverted poorly mixed fluid toward the channel edges, achieving a 100% mixing at all tested flow rates. This strategy was intended to facilitate cost-effective injection-molded lab-on-a-chip devices with enhanced short-range mixing.

Park et al. (contribution 3) developed a microwell-template-assisted magnetic particle trapping technique that avoided the need for microfluidic channels and permitted highly efficient isolation of individual particles. Dual-surface microwell arrays were fabricated on silicon substrates using photolithography, plasma-enhanced chemical vapor deposition, and deep reactive ion etching, yielding hydrophilic well interiors and hydrophobic exteriors. The trapping method integrated magnet-assisted sedimentation with rotational sweeping of a glass slide placed above the microwell array, which realized the rapid and uniform particle trapping across a 250 × 250 well matrix. Trapping performance was characterized using 1.00 μm and 1.88 μm magnetic particles across well dimensions of 1.48, 2.08, and 2.56 μm in width and 1.28, 2.43, and 4.01 μm in depth. Using optimized structures, the trapping efficiency improved with well dimensions, exceeding 93.8% after three operational cycles. Wells with dimensions approximately matching the particle diameter showed high single-particle occupancy. Each trapping cycle could be finished within five minutes. The developed platform was intended to offer a rapid, simple, and scalable route for digital immunoassays and ultrasensitive biomolecular detection.

Valenti et al. (contribution 4) introduced a strategy that employed acousto-mechanical perturbations (AMPs) to modulate cell arrangement and behavior within microchannels. The developed system integrated a smartphone-operated audio speaker with a microfluidic chip, translating sound into mechanical oscillations of the tubing, which then acted upon flow generated by hydrodynamic pressure. Using yeast cells and silica microbeads, they found that acoustic excitation produced periodic changes in flow across varied flow conditions. Yeast cells displayed adjustable responses dependent on flow, whereas inert particles showed only slight, steady modulation. Frequency-domain spectra revealed a dominant response synchronized with the acoustic protocol. This system allowed label-free control of cells and distinguished rigid inert particles from deformable living cells. The approach was intended to offer a flexible, non-invasive, and readily accessible means for controlled cell handling in microfluidic environments.

Wang et al. (contribution 5) reviewed microfluidic and paper-based recombinase polymerase amplification (RPA) systems for decentralized diagnostics and biosurveillance. The review examined the principle and advantages of RPA, its integration with microfluidic technologies, the emergence of paper-based RPA diagnostics, digital microfluidics for quantitative RPA analysis, and intelligent and connected RPA diagnostic systems. Biomedical applications covering respiratory viral infections, reproductive and blood-borne viruses, tuberculosis, bacterial and parasitic diseases, and precision medicine and cancer biomarkers were summarized. Food safety applications including foodborne bacterial pathogens, viral and multiplex detection, aquatic and dairy product safety monitoring, and electrochemical biosensors were discussed, together with environmental monitoring, agricultural and aquatic biosurveillance, and One Health biosurveillance. Challenges related to sample preparation, multiplex stability, quantitative reliability, reagent storage, scalable fabrication, and regulatory validation were analyzed. This review aimed to map the trajectory of RPA-enabled platforms toward accessible molecular diagnostic workflows.

Chang et al. (contribution 6) introduced a microfluidic device for measuring the viscosity of non-Newtonian fluids, in which a high-flow-rate flow-field stabilizer was incorporated to counteract poor flow uniformity at elevated flow rates. Based on a two-phase laminar-flow strategy that derived relative viscosity from channel occupancy, the platform expanded the detectable viscosity range from 1 to 10 cP to 1–50 cP. Computational fluid dynamics (CFD) simulations informed the optimal stabilizer design by adjusting obstacle placement, porosity, and the number of outlet channels. Tests with blood-mimicking and dairy fluids yielded >95% viscosity accuracy and <5% sample-volume deviation relative to commercial viscometers. The chip also resolved viscosity shifts during milk acidification and gelation, showing a good agreement with reference measurements. This platform was designed to advance microfluidic rheometry for food engineering, biomedical diagnostics, and industrial fluid monitoring.

Chitemo et al. (contribution 7) developed a urine-based single-imaging parasite quantification (SIMPAQ) chip for detecting *Schistosoma haematobium* eggs at varying infection loads. Two device configurations were compared by using polystyrene microspheres as surrogates for *Schistosoma haematobium* eggs. The prototype retained particles in the imaging area with 96.00–100% recovery, whereas the second-generation chip, which employed V-shaped micropillars to remove the air-drying step, yielded 95.20–96.00% recovery. With spiked goat urine, efficiencies remained high at 99.33–100%, and the two developed devices successfully trapped real *Schistosoma haematobium* eggs within the imaging areas. This platform was developed to improve diagnostics, disease surveillance, and monitoring for urinary schistosomiasis in resource-limited settings.

Wang et al. (contribution 8) reviewed microfluidic paper-based analytical devices (μPADs) at the edge of real samples, emphasizing recent progress in device architectures, fabrication techniques, substrate selection, assay formats, and practical uses. The review examined fabrication techniques ranging from wax and stencil barriers to laser-defined grooves, 3D-structured multilayers, and inkjet-printed conductive lattices, and analyzed detection strategies including colorimetric, fluorescence, electrochemical, instrument-free distance-encoded, Raman/SERS-based, luminescent, and mass spectrometry-assisted readouts. Applications in human biofluid diagnosis, food safety analysis, environmental monitoring, and pesticide and illicit drug testing were summarized. The review also discussed artificial intelligence (AI)-assisted design optimization, signal processing, and data interpretation, and identified critical challenges related to material heterogeneity, signal instability, matrix interference, and scalable manufacturing. This perspective aimed to clarify how paper-based platforms could move toward reliable decentralized sensing and translational deployment.

Chen et al. (contribution 9) reported a cost-effective, high-resolution fabrication strategy forμPADs, termed wax soft lithography, which integrated wax printing with soft lithography. The strategy used photolithography to fabricate a master mold, followed by polydimethylsiloxane (PDMS) molding to replicate the chip, which was sealed with a PDMS film to retain molten wax within the microchannels. After heating at 75 °C for 150 s with a 6 kg weight applied, the wax migrated from the channels onto the paper, thereby generating the desired patterns. Channels with a smallest width of 234 ± 62 μm were reproducibly obtained and intricate designs could be successfully patterned. The as-prepared μPADs were applied to measure glucose in glucose-spiked urine, yielding a colorimetric readout that depended on concentration, with the M-channel signal strengthening as glucose levels increased. This approach was designed to offer an efficient route for rapidly fabricating high-resolution μPADs for point-of-care testing in resource-constrained settings.

Pan et al. (contribution 10) reviewed the recent advances on female reproductive organ-on-a-chip (OOC) platforms, covering ovary-, uterus-, placental- and fallopian tube- and multi-organ-on-a-chip systems. The review examined fabrication methods, material selection, and physiological and anatomical simulation strategies. Applications in assisted reproduction, hormonal regulation, embryo–endometrium interaction, endometriosis, gynecologic cancers, and drug screening were summarized. The review further discussed multi-organ integration for systemic reproductive modeling, incorporation of microbiome interactions, and clinical translation for drug action mechanisms. Key challenges including primary cell sourcing, long-term culture stability, PDMS absorption of hydrophobic compounds, protocol standardization, and scalability for high-throughput screening were analyzed. This review aimed to provide technical insights for reproductive biology and medicine and to support the development of physiologically relevant models for drug development and toxicity assessment.

Sabahi-Kaviani et al. (contribution 11) fabricated a nervous system-on-chip (NoC) by combining microfabrication with neurons obtained from human induced pluripotent stem cells (hiPSCs). Microtunnel devices (MDs) were produced in radial and linear geometries to enable compartmentalized cultivation of cortical and enteric neural networks. These MDs supported axonal extension while preventing somata and dendrites from translocating between compartments, thereby encouraging the assembly of organized neuronal networks. The resulting cultures retained viability, displayed lineage-associated markers such as MAP2, Tau, NF200, and Synapsin, and exhibited synaptic formation. With tunnel dimensions of 10 μm in width and 4 μm in height, cell bodies were excluded from the tunnels, so only axons could reach neighboring wells. The platform was also applied to probe how sealing substrate composition and surface topography modulated enteric neuronal differentiation. This system was intended to serve as a microfluidic tool for exploring human neural network biology and neurodegenerative disorders.

Wu et al. (contribution 12) developed a four-channel microfluidic vascular-wall chip to recreate the endothelial–extracellular matrix–smooth muscle cell architecture and to model early atherosclerotic inflammatory endothelial dysfunction. The device incorporated a perfusable endothelial channel, a collagen I hydrogel compartment containing human aortic smooth muscle cells, an acellular matrix zone, and a medium supply channel. Under the physiological conditions, human umbilical vein endothelial cells (HUVECs) established a ZO-1-expressing endothelial barrier and retained high viability. Exposure to TNF-α and IL-1β (10 ng/mL each) increased IL-6 release, enhanced the recruitment of THP-1-derived M0-like macrophages, compromised ZO-1 continuity, and raised FITC-dextran permeability, without inducing extensive cell death. The chip was then employed to assess metformin and atorvastatin and the combination attenuated MCP-1 secretion more effectively than either agent alone. This platform was intended to offer a microfluidic model of early inflammatory endothelial dysfunction for mechanistic studies and preliminary anti-inflammatory drug evaluation.

Sabahi-Kaviani et al. (contribution 13) established a nanofabrication method that combined photolithographic patterning with microtransfer molding to generate nanogrooves from the photocurable resin NOA81 on microelectrode array (MEA) substrates. Scanning electron microscopy and atomic force microscopy confirmed that the resulting nanogrooves possessed a spatial periodicity of 976 ± 16 nm, a ridge linewidth of 232 ± 6 nm, and a ridge height of 68.2 ± 0.9 nm. Human induced pluripotent stem cell-derived Ngn2 iNeurons were then seeded onto the nanogroove-functionalized MEAs to evaluate biocompatibility and functional effects. These neurons displayed neurites aligned along the groove axis and exhibited pronounced spiking activity together with increased burst frequency relative to randomly oriented neuronal cultures. This fabrication method was intended to improve spike activity and network organization for neural models with greater in vivo resemblance.

Tang et al. (contribution 14) reviewed the integration of AI and microfluidics for drug delivery applications, focusing on the transition from empirical, trial-and-error formulation toward data-driven and automated workflows. The review examined microfluidic architectures including microfluidic hydrodynamic focusing, chaotic mixing, and droplet-based systems, together with AI toolsets such as supervised learning, unsupervised learning, reinforcement learning, deep learning, and Bayesian optimization. It further discussed AI-enhanced synthesis of lipid nanoparticles, liposomes, polymeric nanoparticles, hydrogels, and solid lipid nanoparticles, as well as AI-assisted phenotypic screening using high-content screening and organ-on-a-chip models. Regulatory and practical challenges, including data scarcity, scalability, material compatibility, explainability, and the FDA risk-based credibility assessment framework, were analyzed. This review aimed to bridge the physical–digital divide and provide a roadmap toward self-driving laboratories in precision nanomedicine.

It is anticipated that the emerging technologies and novel innovations in microfluidics will exert transformative effects on biomedical research.
